# The Free Achilles Tendon Is Shorter, Stiffer, Has Larger Cross-Sectional Area and Longer T2^*^ Relaxation Time in Trained Middle-Distance Runners Compared to Healthy Controls

**DOI:** 10.3389/fphys.2020.00965

**Published:** 2020-08-27

**Authors:** Daniel Devaprakash, Steven J. Obst, David G. Lloyd, Rod S. Barrett, Ben Kennedy, Iain Ball, Kahlee L. Adams, Tyler J. Collings, Giorgio Davico, Adam Hunter, Nicole Vlahovich, David L. Pease, Claudio Pizzolato

**Affiliations:** ^1^ School of Allied Health Sciences, Griffith University, Southport, QLD, Australia; ^2^ Griffith Centre for Biomedical and Rehabilitation Engineering, Menzies Health Institute Queensland, Griffith University, Gold Coast, QLD, Australia; ^3^ School of Health, Medical, and Applied Sciences, Central Queensland University, Bundaberg, QLD, Australia; ^4^ QSCAN Radiology Clinics, Gold Coast, QLD, Australia; ^5^ Philips Healthcare, Australia and New Zealand, Sydney, NSW, Australia; ^6^ Australian Institute of Sport, Canberra, ACT, Australia; ^7^ Department of Industrial Engineering, Alma Mater Studiorum-University of Bologna, Bologna, Italy; ^8^ Medical Technology Lab, IRCCS Istituto Ortopedico Rizzoli, Bologna, Italy

**Keywords:** free Achilles tendon, magnetic resonance imaging, freehand three-dimensional ultrasound, trained middle-distance runners, geometry, Young’s modulus, T2^*^ relaxation time

## Abstract

Tendon geometry and tissue properties are important determinants of tendon function and injury risk and are altered in response to ageing, disease, and physical activity levels. The purpose of this study was to compare free Achilles tendon geometry and mechanical properties between trained elite/sub-elite middle-distance runners and a healthy control group. Magnetic resonance imaging (MRI) was used to measure free Achilles tendon volume, length, average cross-sectional area (CSA), regional CSA, moment arm, and T2^*^ relaxation time at rest, while freehand three-dimensional ultrasound (3DUS) was used to quantify free Achilles tendon mechanical stiffness, Young’s modulus, and length normalised mechanical stiffness. The free Achilles tendon in trained runners was significantly shorter and the average and regional CSA (distal end) were significantly larger compared to the control group. Mechanical stiffness of the free Achilles tendon was also significantly higher in trained runners compared to controls, which was explained by the group differences in tendon CSA and length. T2^*^ relaxation time was significantly longer in trained middle-distance runners when compared to healthy controls. There was no relationship between T2^*^ relaxation time and Young’s modulus. The longer T2^*^ relaxation time in trained runners may be indicative of accumulated damage, disorganised collagen, and increased water content in the free Achilles tendon. A short free Achilles tendon with large CSA and higher mechanical stiffness may enable trained runners to rapidly transfer high muscle forces and possibly reduce the risk of tendon damage from mechanical fatigue.

## Introduction

The Achilles tendon stores and recovers strain energy to improve mechanical energy generation-absorption of the triceps surae muscles and reduce metabolic cost during dynamic activities (Alexander and Bennet-Clark, 1977; Roberts et al., 1997; Lichtwark and Wilson, 2005; Wiesinger et al., 2017). *In vivo* studies have shown that Achilles tendon geometry and/or mechanical properties adapt to long-term mechanical loading in a manner that is specific to the type and duration of the applied loads (Bohm et al., 2015; Wiesinger et al., 2015, 2016). These long-term adaptations are believed to enhance the mechanical function of the Achilles tendon and triceps surae complex and keep the strain experienced by the Achilles tendon within physiological limits (Ker et al., 1988; Bohm et al., 2015; Wiesinger et al., 2015, 2016).

Rosager et al. (2002), Magnusson and Kjaer (2003), and Wiesinger et al. (2016) reported 15, 30, and 36% larger non-normalised cross-sectional area (CSA) in male runners compared to non-runners, respectively. Similar differences are also reported between runners and non-weight bearing athletes (e.g., kayakers; Kongsgaard et al., 2005), and in older endurance runners versus older non-runners (+16%) and younger non-runners (+30%; Stenroth et al., 2016). While free Achilles tendon CSA tends to be higher in habitual runners (Rosager et al., 2002; Magnusson and Kjaer, 2003), a recent review suggests there is limited evidence to support a concomitant increase in Achilles tendon mechanical stiffness and/or Young’s modulus (Wiesinger et al., 2015). However, unlike CSA, which is commonly measured from free Achilles tendon region, mechanical properties are often measured from the gastrocnemius muscle-tendon junction to the calcaneus (Lichtwark and Wilson, 2005; Bohm et al., 2014; Wiesinger et al., 2016), which also includes the aponeurosis. Thus, understanding the adaptation of the free Achilles tendon requires both geometry and mechanical properties of the free portion of the tendon (from the soleus muscle-tendon junction to the calcaneus) to be assessed independently of the aponeurosis. While magnetic resonance imaging (MRI) and freehand three-dimensional ultrasound (3DUS) methods have been used to assess resting tendon geometry (Devaprakash et al., 2019), freehand 3DUS has the added advantage over MRI of allowing tendon geometry to be assessed in the laboratory environment at rest and under load (Obst et al., 2014a; Nuri et al., 2017), so that the mechanical properties of the tendon can also be evaluated.

Ultrashort echo time (UTE) based MRI methods have been used to capture average T2^*^ relaxation time of the Achilles tendon (mid-portion, insertion region, muscle-tendon junction, and whole Achilles tendon-aponeurosis) in a group of recreational long distance runners compared to healthy volunteers, showing longer T2^*^ relaxation times in the mid-portion and whole Achilles tendon-aponeurosis for runners (Grosse et al., 2015). Longer T2^*^ relaxation times are believed to reflect a greater amount of free water protons between collagen fibres, reduced collagen alignment, and have previously been reported to distinguish between healthy and pathological Achilles tendons (Juras et al., 2012, 2013; Gärdin et al., 2016; Qiao et al., 2017). However, it remains unclear if T2^*^ relaxation time, which is indicative of collagen organisation/disorganisation (Juras et al., 2013; Gärdin et al., 2016), is related to Young’s modulus in the free Achilles tendon. Any such relationship would be expected to depend on the sensitivity of T2^*^ relaxation time to fluid alterations compared to alterations in collagen disorganisation, as well as the extent to which collagen disorganisation is sufficient to influence the bulk tissue modulus.

The purpose of this study was to use MRI and freehand 3DUS to: (1) compare free Achilles tendon geometry, mechanical stiffness, Young’s modulus, length normalised mechanical stiffness, and T2^*^ relaxation time between trained elite/sub-elite middle-distance runners and a healthy control group; and (2) determine the relationship between T2^*^ relaxation time and Young’s modulus of the free Achilles tendon. Given the strong existing evidence showing that the free Achilles tendon CSA of runners tends to be higher than non-runners (Rosager et al., 2002; Magnusson and Kjaer, 2003; Wiesinger et al., 2016), it was hypothesised that trained runners would have a larger free Achilles tendon CSA resulting in greater free Achilles tendon mechanical stiffness. Based on the findings of Grosse et al. (2015), T2^*^ relaxation times were also expected to be higher in runners compared to a healthy control group.

## Materials and Methods

### Participant Characteristics

Sixteen trained elite/sub-elite middle-distance runners (10 males and 6 females) and 16 healthy controls (11 males and 5 females) with no prior history or symptoms of Achilles tendon injury participated in the study. Trained runners performed more than 80 km of running per week as part of their training, competed regularly at state/national/international level competitions, and had no recent or recurrent Achilles tendon or lower limb injuries as assessed by an experienced physician. Trained runners with symptomatic Achilles tendinopathy as determined via clinical assessment (Maffulli et al., 2003; Scott et al., 2020) and ultrasound imaging (brightness mode and microbubble technique) were excluded. The study was approved by the relevant institutional Human Research Ethics Committees and all participants provided written informed consent prior to participation in the study.

### Experimental Design and Data Collection Protocol

Data collection was performed at two sites (Trained runners: Australian Institute of Sport, Canberra, Australia; Healthy controls: Griffith University, Queensland, Australia) due to the inability to test participants from both groups at a single testing centre. Participants attended two experimental testing sessions. During the first session, MRI scans (anatomical and UTE T2^*^ relaxation time) of the participants’ free Achilles tendon were obtained (Trained runners: Universal Medical Imaging, ACT, Australia; Healthy controls: QSCAN Radiology Clinics, Queensland, Australia). In the second session, freehand 3DUS scans were performed at rest and under load in order to determine free Achilles tendon mechanical stiffness, Young’s modulus, and length normalised mechanical stiffness. The freehand 3DUS set-ups at the two experimental testing sites were identical. Participants refrained from strenuous physical activity in the 24 h before each experimental session. Prior to each session, all participants completed a preconditioning protocol that involved walking at a self-selected pace (~270 gait cycles; Hawkins et al., 2009). All measures were obtained from the participants’ preferred/dominant leg. Free Achilles tendon geometry of trained middle-distance runners recorded during rest using MRI and freehand 3DUS has been partly presented in Devaprakash et al. (2019).

### Magnetic Resonance Imaging

Anatomical MRI scans of the distal lower limb (i.e., including distal tibia-fibula and foot) were acquired on a Philips Ingenia 3.0 Tesla scanner (Ingenia 3.0 T, MR system, Philips medical systems, Amsterdam, North Holland, Netherlands) using an eight-channel ankle coil (ACT, Queensland: PDW 3D TSE, TR/TE 1000/41 ms) with the participant lying in a supine position with the hip in neutral position, the knee fully extended, and the ankle in a neutral position (0° dorsiflexion). Slice thickness, slice gap, and resolution were 0.6, 0.3, and 0.27 mm, respectively. MRI scans were acquired in the sagittal plane and exported in DICOM format for further processing.

With the participant’s ankle positioned at approximately 30° plantarflexion, UTE T2^*^ relaxation time sequence (Fast Field Echo) was acquired using a 16-channel knee coil to assess tissue quality. Imaging parameters for obtaining UTE T2^*^ data at both MRI centres: (1) relaxation time (TR; ACT, Queensland: 14.674 and 16.481 ms), (2) echo time (TE; ACT, Queensland: 0.18, 2.50, 4.82, 7.14, 9.46 and 0.21, 1.86, 3.51, 5.15, 6.80, 8.45, 10.09 ms), (3) flip angle (ACT, Queensland: 10°), and (4) image matrix (ACT, Queensland: 256 × 256). UTE T2^*^ sequence data were exported in PAR/REC (Queensland) and XML/REC (ACT) formats.

**Figure 1:**
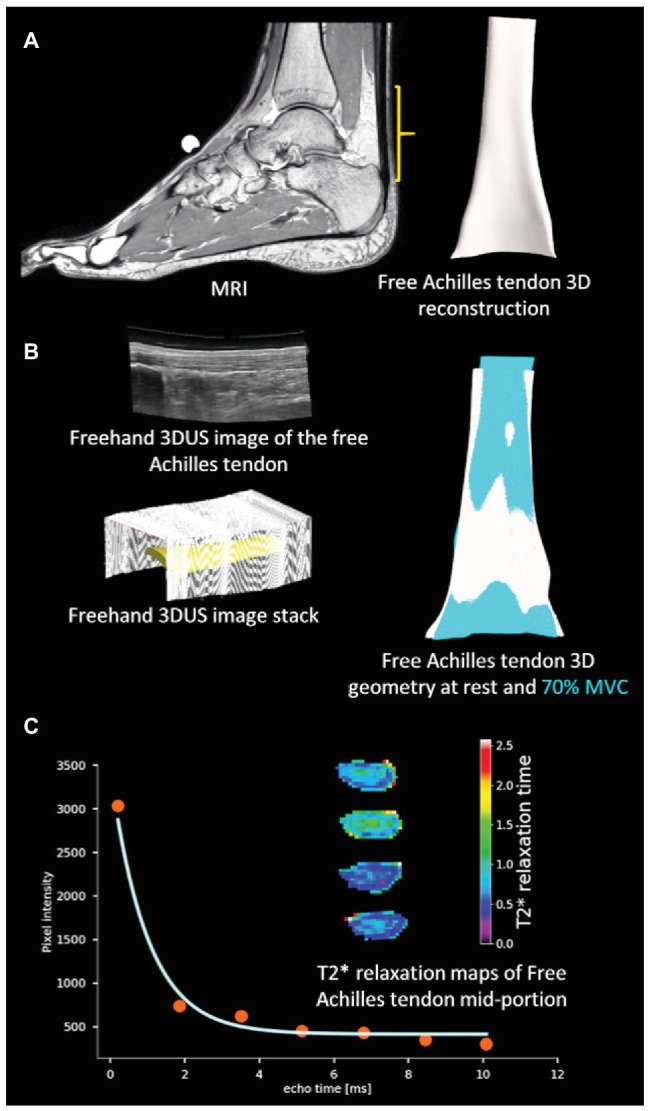
Overview of methodology: **(A)** three-dimensional (3D) reconstruction of the free Achilles tendon from MRI **(B)** 3D ultrasound (3DUS) reconstruction of free Achilles tendon from freehand 3DUS images and free Achilles tendon geometry during rest and active muscle contraction condition [70% maximal voluntary contraction (MVC)], and **(C)** T2^*^ relaxation time of a sample pixel in the free Achilles tendon by fitting an exponentially decaying curve, and sample colour maps of image slices of the free Achilles tendon mid-portion.

Anatomical MRI data stored in DICOM format were converted to Stradwin files (version 5.4, Medical imaging group, University of Cambridge, Cambridge, England, UK). Following this, MRI data were resliced at one-pixel resolution (0.27 mm) in the transverse plane (Treece et al., 2003) and sparse manual segmentation of the free Achilles tendon was performed by the same experimenter (DD, unblinded to the participant’s identity) in the transverse plane at uniform space intervals (6–8 slices per tendon) for all participants. Free Achilles tendon insertion at the calcaneal notch and the soleus muscle-tendon junction were defined as distal and proximal end of the free Achilles tendon. Free Achilles tendon 3D reconstruction was performed using sparse contour interpolation method (Treece et al., 2000; Figure 1A). Additionally, volume, regional CSA, and 3D centroid location of each slice of the free Achilles tendon were exported in csv format. Tendon length was calculated as the cumulative sum of the distance between the centroid of consecutive slices (Obst et al., 2014a). Average CSA of the free Achilles tendon was calculated by dividing volume by length. Free Achilles tendon geometry obtained from anatomical MRI scans was used to report free Achilles tendon geometry (volume, length, average, and regional CSA) in the trained runners and healthy controls group.

The moment arm of the free Achilles tendon was measured from the anatomical MRI scans acquired from all the participants with the ankle positioned at 0° dorsiflexion. A sparse manual segmentation of the talus was performed in the transverse plane. Sparse contour interpolation method was used to reconstruct the talus 3D surface. Following this, a cylinder was fit to the dome of the talus using in-built MATLAB functions (Torr and Zisserman, 2000) and visually inspected for correctness. The points defining the axis and centroid of the cylinder were exported and a cubic spline interpolation was performed. Similarly, a cubic spline interpolation of the centroid points of each slice of the free Achilles tendon was also performed. The shortest distance between the two interpolated curves (i.e., axis of the cylinder and centroid line of the free Achilles tendon) was obtained using n-dimensional nearest point search method (Barber et al., 1996) and was defined as the free Achilles tendon moment arm (Alexander et al., 2017).

An open source python module (Millman and Brett, 2007; Nipy/Nibabel: https://doi.org/10.5281/zenodo.1287921) was used to parse PAR/REC files while custom MATLAB scripts were used to parse XML/REC files. T2^*^ relaxation time of each pixel was obtained by fitting a mono-exponential decay curve with bias to the pixel values measured at different echo times using Levenberg-Marquardt algorithm implemented in open source python module (Newville et al., 2016; *lmfit*: https://doi.org/10.5281/zenodo.2620617; Figure 1C). In the first step, the exponential model available in *lmfit* was used to estimate the initial parameters. Following this step, a custom exponential model accounting for bias was created and the parameters estimated in the first step were used to estimate the T2^*^ relaxation time. A manual segmentation of the free Achilles tendon cross-section was performed to extract individual T2^*^ relaxation time values and the average T2^*^ relaxation time from the cropped pixels was obtained for each participant.

### Freehand 3DUS

All freehand 3DUS data were acquired, processed, and analysed by the same experimenter (DD) to quantify free Achilles tendon mechanical stiffness, Young’s modulus, and length normalised mechanical stiffness. 2D ultrasound (Aplio 500, Canon Medical Systems Corporation, Otawara, Tochigi, Japan) and motion capture (Vicon MX T series, Vicon Motion Systems Ltd., Oxford, England, UK) data were acquired at 30 and 100 Hz, respectively. The ultrasound transducer (PLT-805AT, linear probe, width: 58 mm, frequency range: 5–12 MHz, Canon Medical Systems Corporation, Otawara, Tochigi, Japan) was fitted with six rigidly attached retroreflective markers. A pulse signal generated via an external trigger was used to synchronise 2D ultrasound and motion capture data. The ultrasound imaging parameters were kept constant at both experimental test centres (10 MHz, depth: 30 mm, resolution: 0.063 mm, capture frequency: 30 Hz, acoustic power: 100%, and mechanical index: 1.5). Prior to acquisition, temporal and spatial calibration of the ultrasound transducer was performed in a water bath (35°C) using a single wall phantom procedure (Prager et al., 1998). Visualisation of the free Achilles tendon cross-section was enhanced by securely attaching an acoustic standoff pad (~20 mm) to the transducer during data acquisition. A thin layer of ultrasound transmission gel (Other-sonic, Pharmaceutical Innovations, Newark, NJ, USA) was applied to the participant’s skin to ensure smooth movement of the ultrasound transducer. Participants were positioned prone on a bed with their foot firmly secured to a foot plate with in-built dynamometer (Australian Institute of Sport: HUMAC NORM, Stoughton, MA, USA, Griffith University: Futek TFF600, Irvine, California, USA). The foot plate was locked with the ankle in a neutral position. The knee joint was fully extended and the hip joint was in neutral position (i.e., participant laid flat on the dynamometer bed).

### Tendon Mechanical Testing

Free Achilles tendon mechanical stiffness, Young’s modulus, and length normalised mechanical stiffness were obtained using a staged isometric plantarflexion contraction protocol (Peltonen et al., 2010). Freehand 3DUS images of the free Achilles tendon were acquired using a single transverse sweep (Figure 1B) at rest and three submaximal isometric loading conditions (25 ± 5, 50 ± 5, and 70 ± 5% of maximal voluntary isometric plantarflexion torque at neutral ankle angle). A minimum of three trials were obtained for each loading condition. Maximal voluntary isometric plantarflexion torque was obtained prior to tendon testing using the peak torque value from three 3–5 s ramped contractions. Visual feedback was used to ensure each target torque was reached and maintained throughout each stage. Scan duration was 15–25 s for the resting scans and 10–15 s during the submaximal contractions. Recorded data were discarded if the participant was unable to maintain the target torque within the specified range during submaximal isometric loading conditions.

All freehand 3DUS data were resliced at one-pixel resolution using Stradwin to ensure that errors due to misidentification of most proximal and distal slice of the free Achilles tendon were minimised (Devaprakash et al., 2019). Segmentation steps performed on MRI data were repeated on freehand 3DUS data. The calcaneal notch of four trained runners could not be imaged as the dynamometer foot plate heel support partially covered the participant’s calcaneus. In these four instances, the most distal part of the calcaneus visible in 3DUS scan was identified and used as common starting point for the segmentation of the free Achilles tendon. The free Achilles tendon of each participant was segmented at each of the four different loading conditions. Tendon force at each condition was calculated by dividing the experimental ankle torque with the tendon moment arm obtained from the anatomical MRI. The experimental ankle torque represented the measured net ankle torque under load minus the measured net ankle torque at rest. Mechanical stiffness (N.mm^−1^) of the free Achilles tendon was calculated by obtaining the slope of the line fitted to the linear region of the force-elongation data measured at 25, 50, and 70% active muscle contraction conditions. Young’s modulus (GPa) of the free Achilles tendon was calculated by obtaining the slope of the line fitted to the linear region of the tendon stress-strain curve measured at 25, 50, and 70% active muscle contraction conditions. Length normalised mechanical stiffness (kN/strain) was calculated as the product of free Achilles tendon mechanical stiffness and resting length (Mersmann et al., 2017). Free Achilles tendon length calculated from freehand 3DUS data during resting scan was used as a reference measure in deformation and strain calculations. Tendon stress was calculated as tendon force divided by the average CSA of the free Achilles tendon measured during the active muscle contraction condition, and as such, represented the “true” tendon stress (Obst et al., 2014b). Tendon strain (%) was calculated as the change in length compared to resting length divided by the resting length.

### Statistical Analysis

Analysis of variance (ANOVA) was used to assess the effect of group (trained runners versus healthy controls) on participant characteristics, Achilles tendon geometry, mechanical stiffness, Young’s modulus (including parameters used to derive stiffness and modulus), and length normalised mechanical stiffness. A Kruskal-Wallis H-test was used to assess group differences in T2^*^ relaxation time. All statistical analyses were performed using an open source python module (Vallat, 2018; Pingouin: https://doi.org/10.5281/zenodo.3386497). Additionally, statistical parametric mapping independent sample *t*-test (Pataky, 2012) was used to assess group differences in regional CSA measured at 1% intervals across the full length of the free Achilles tendon. Prior to conducting the statistical tests, all data were assessed for normality using Shapiro-Wilk test. Statistical parametric mapping test was conducted using an open source python module (spm1d: http://www.spm1d.org/). The relationship between T2^*^ relaxation time and Young’s modulus was reported using Pearson’s product moment correlation coefficient.

## Results

**Figure 2:**
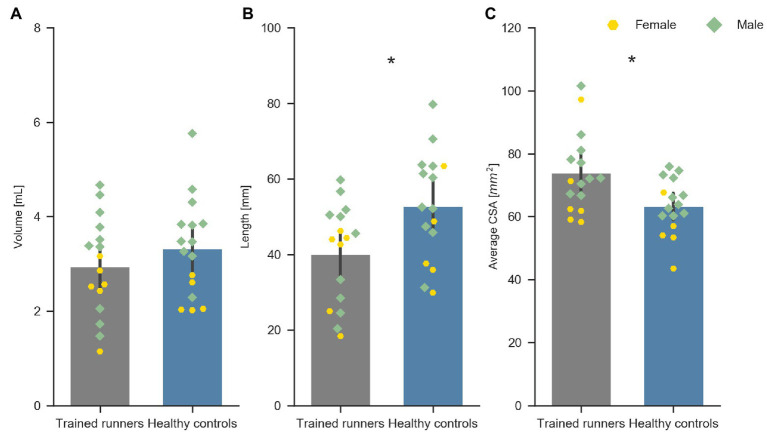
Volume **(A)**, length **(B)**, and average CSA **(C)** of the free Achilles tendon of trained middle-distance runners (*n* = 16) and healthy control group (*n* = 16). *Indicates significant difference between the two groups.

**Figure 3:**
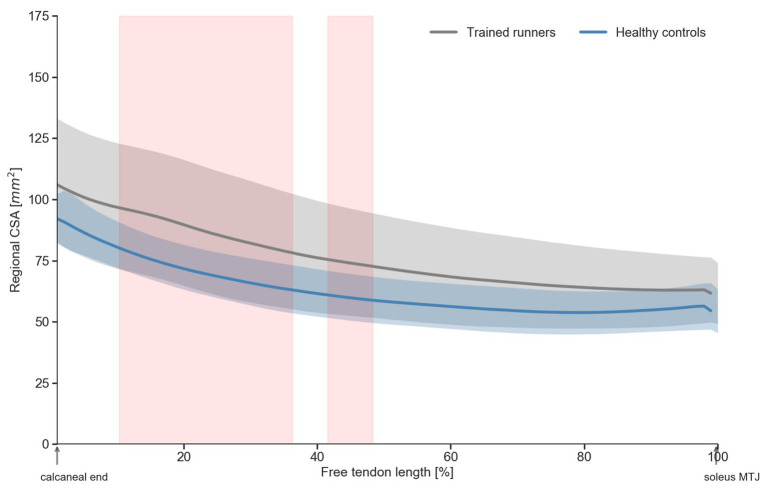
Regional cross-sectional area (CSA) of the free Achilles tendon of trained middle-distance runners and healthy control group. Shaded regions (in red) indicate significant (*p*>0.05) difference in regional CSA between trained middle-distance runners and healthy control group.

The healthy controls were significantly older and had higher body mass index than the trained runners but were not different in height, body mass, or tibial length (Table 1). Volume of the free Achilles tendon was not significantly different between trained runners and healthy controls (*F*_1, 30_ = 1.071, *p* = 0.31, *η* ^2^ = 0.03; Figure 2A). Length of the free Achilles tendon was significantly shorter in trained runners compared to healthy controls (*F*_1, 30_ = 6.675, *p* = 0.01, *η* ^2^ = 0.18; Figure 2B). Average CSA of the free Achilles tendon was significantly larger in trained runners compared to healthy controls (*F*_1, 30_ = 7.662, *p* = 0.009, *η* ^2^ = 0.20; Figure 2C). Regional CSA of the free Achilles tendon in the distal region to mid region was also significantly larger in trained runners (Figure 3). There was no significant group difference in tendon moment arm (Table 1, *F*_1, 30_ = 0.578, *p* = 0.45, *η* ^2^ = 0.02).

**Table 1:** Participant characteristics by group.

	Runners	Controls	F, *p*
Age (years)	25.2 ± 5.0	30.3 ± 4.9	8.6, 0.01
Height (cm)	175.5 ± 7.3	172.4 ± 10.5	0.9, 0.35
Body mass (kg)	64.4 ± 8.4	71 ± 16.8	2.0, 0.20
Body mass index (kg m^−2^)	20.9 ± 1.8	23.8 ± 4.5	5.7, 0.02
Tibial length (cm)	41.6 ± 2.2	41.3 ± 3.1	0.11, 0.74
Achilles tendon moment arm (cm)	5.0 ± 0.3	4.9 ± 0.5	0.462, 0.50

Data are mean ± one standard deviation.

**Figure 4:**
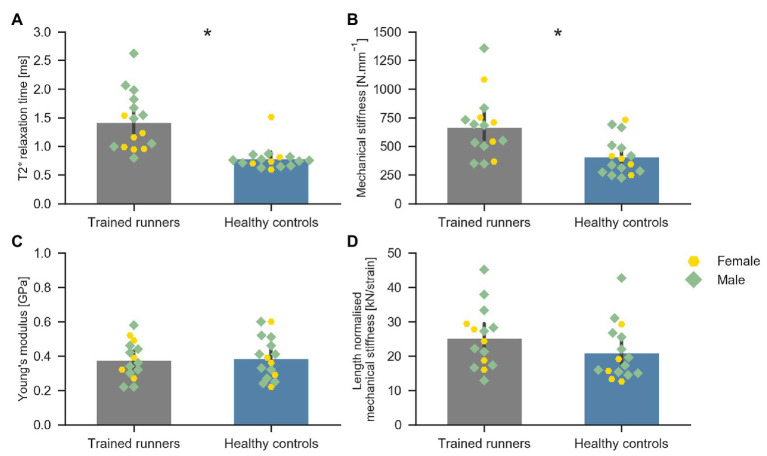
Average T2^*^ relaxation time of the free Achilles tendon of trained middle-distance runners (*n* = 16) and healthy control group (*n* = 16) **(A)**, Mechanical stiffness (N mm^−1^) of the free Achilles tendon of trained middle-distance runners (*n* = 15) and healthy control group (*n* = 16) **(B)**, Young’s modulus of the free Achilles tendon of trained middle-distance runners (*n* = 15) and healthy control group (*n* = 16) **(C)**, and length normalised mechanical stiffness of the free Achilles tendon of trained middle-distance runners (*n* = 15) and healthy control group (*n* = 16) **(D)**. *Indicates significant difference between the two groups.

**Figure 5:**
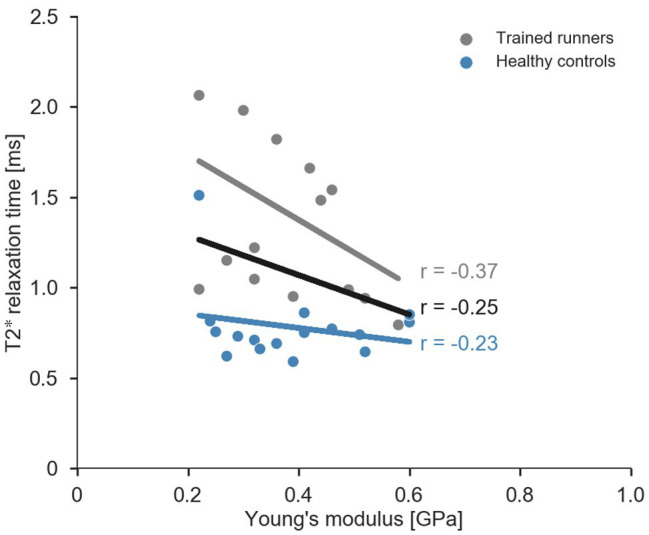
Scatterplot and regression lines (combined data: black, trained runners: grey, and healthy controls: blue) for relationship between average T2^*^ relaxation time and Young’s modulus of the free Achilles tendon.

Average T2^*^ relaxation time of the free Achilles tendon was significantly longer in trained runners compared to healthy controls [*H*(1) = 18.876, *p* < 0.001, *η* ^2^ = 0.59, Figure 4A]. Mechanical stiffness of the free Achilles tendon was significantly higher in trained runners (*F*_1, 29_ = 10.198, *p* = 0.003, *η* ^2^ = 0.26, Figure 4B), however Young’s modulus (*F*_1, 29_ = 0.053, *p* = 0.71, *η* ^2^ = 0.002, Figure 4C) and length normalised mechanical stiffness (*F*_1, 29_ = 1.933, *p* = 0.17, *η* ^2^ = 0.062, Figure 4D) were similar between the two groups. Maximum torque (runners: 95.1 ± 21.4 Nm, controls: 81.7 ± 19.9 Nm, *F*_1, 29_ = 3.261, *p* = 0.08, *η* ^2^ = 0.101) and maximum force (runners: 1,898 ± 377 N, controls: 1,664 ± 281 N, *F*_1, 29_ = 3.861, *p* = 0.06, *η* ^2^ = 0.118) recorded during tendon mechanical testing session were similar between the two groups. Torque, force, elongation, stress, and strain used to calculate mechanical stiffness and Young’s modulus of the free Achilles tendon are reported in Table 2. Force measured during 70% maximal voluntary contraction (MVC) condition was significantly higher in trained runners when compared to the healthy control group (*F*_1, 29_ = 4.262, *p* = 0.05, *η* ^2^ = 0.13, Table 2). Free Achilles tendon elongation measured during 70% MVC was significantly higher in the healthy control group when compared to trained runners (*F*_1, 29_ = 5.363, *p* = 0.03, *η* ^2^ = 0.16, Table 2). During 25% MVC, free Achilles tendon stress was significantly higher in healthy controls compared to trained runners (*F*_1, 29_ = 7.018, *p* = 0.01, *η* ^2^ = 0.20, Table 2). The Pearson’s product moment correlation coefficient between T2^*^ relaxation time and Young’s modulus were −0.25 for all participants (*p* = 0.16), −0.37 for trained runners (*p* = 0.18), and −0.23 for healthy controls (*p* = 0.40; Figure 5).

**Table 2:** Torque, force, elongation, stress, and strain data recorded during free Achilles tendon mechanical testing using freehand three-dimensional ultrasound.

	25% MVC		50% MVC		70% MVC	
	Runners	Controls	Runners	Controls	Runners	Controls
Torque (Nm)	22.8 ± 5.8	22.2 ± 5.7	47.3 ± 10.6	41.0 ± 9.5	67.0 ± 15.2	56.4 ± 14.6
Force (N)	456 ± 105	453 ± 85	943 ± 188	837 ± 131	1,337 ± 266^*^	1,158 ± 214
Elongation (mm)	0.77 ± 0.31	0.88 ± 0.42	1.48 ± 0.35	1.84 ± 0.63	2.07 ± 0.62	2.63 ± 0.71^*^
Stress (MPa)	6.38 ± 0.94	7.62 ± 1.56^*^	13.25 ± 2.29	14.37 ± 2.62	19.66 ± 2.57	20.22 ± 4.01
Strain (%)	1.92 ± 0.59	1.65 ± 0.67	3.82 ± 0.62	3.38 ± 0.98	5.26 ± 0.59	4.94 ± 1.08

Data are mean ± one standard deviation.^*^Indicates significant difference between runners and healthy controls.

## Discussion

This is the first study to comprehensively report geometry and mechanical properties of the free Achilles tendon of trained elite/sub-elite middle-distance runners. Consistent with our hypothesis, the free Achilles tendons of trained elite/sub-elite middle-distance runners were stiffer due to an altered geometry. We also found that T2^*^ relaxation time was longer in trained runners, which is believed to be indicative, at least in part, of collagen disorganisation (Juras et al., 2013; Gärdin et al., 2016). The finding that T2^*^ relaxation time was not significantly correlated with Young’s modulus suggests that T2^*^ relaxation time should not be used to infer free Achilles tendon material properties.

The average resting free Achilles tendon CSA was ~16% larger in trained middle-distance runners compared to healthy controls, which is broadly consistent with reports of a 15% higher CSA in endurance runners compared to healthy controls (Wiesinger et al., 2016), although not as high as the 30–36% larger CSA in runners compared to healthy controls and non-weight bearing athletes reported by Rosager et al. (2002) and Kongsgaard et al. (2005), respectively. We also found that the free tendon CSA of runners was significantly larger at the distal (calcaneal) end when compared with healthy controls. We also observed an increase in tendon CSA along the length of the free Achilles tendon (i.e., proximal end to distal end), in both runners and healthy controls consistent with a prior report in an asymptomatic population (Obst et al., 2014b). A significantly larger free Achilles tendon CSA compared to healthy controls likely reflects a positive adaptation for runners resulting in higher free Achilles tendon stiffness and concomitant reduction in the peak strain experienced during dynamic activities, as well as mitigation of mechanical fatigue (Buchanan and Marsh, 2001).

The free Achilles tendon length was significantly shorter in trained middle-distance runners compared to healthy controls, and as such, we found no difference in free Achilles tendon volume between the two groups. A short and stiff tendon with larger CSA may represent a favourable adaptation for short- and middle-distance runners, as this enables the rapid transfer of greater triceps surae muscle force to the calcaneal insertion (Biewener and Roberts, 2000; Roberts, 2002). However, it is difficult to determine whether this in fact reflects an adaptation due to training or represents a preferred phenotype for middle-distance runners. In this context, it is plausible that in middle-distance runners, force production may be favoured over metabolic power consumption, as a stiffer free Achilles tendon would enable more direct transmission of muscle force (Roberts, 2002). Further, since we did not measure the whole Achilles tendon geometry (i.e., free Achilles tendon and aponeurosis), we do not know what influence a short free Achilles tendon would have on the mechanical behaviour of the whole Achilles tendon-aponeurosis. In the present study, we decided to present absolute measurements of free Achilles tendon geometry rather than normalised values as our findings did not change when normalising free Achilles tendon CSA to participant mass or free Achilles tendon length to tibial length.

The mechanical stiffness of the free Achilles tendon was 47% higher in trained runners compared to healthy controls. Our estimates of free Achilles tendon stiffness (trained runners = 667 ± 275 N.mm^−1^ and healthy controls = 410 ± 164 N.mm^−1^) are similar to those reported for free Achilles tendon by Magnusson et al. (2003) (759 ± 132 N.mm^−1^); however, the mechanical stiffness values we have reported are greater than the values reported by other studies (Rosager et al., 2002; Arampatzis et al., 2010; Arya and Kulig, 2010) for the whole Achilles tendon-aponeurosis system. The mechanical stiffness reported by Kongsgaard et al. (2011) for the free Achilles tendon (2,622 ± 534 N.mm^−1^) are almost four times greater than the values reported in the present study. Our estimates of Young’s modulus (trained runners = 0.38 ± 0.11 GPa and healthy controls = 0.39 ± 0.12 GPa) were close to the lower end of Young’s modulus values reported by *in vivo* (0.78 ± 0.18 GPa; Magnusson et al., 2003) and *in vitro* (0.38 ± 0.10 GPa; Lewis and Shaw, 1997; 0.82 ± 0.21 GPa; Wren et al., 2001) studies of the free Achilles tendon. The wide variation in mechanical stiffness and Young’s modulus values reported in the literature may be due to differences in the portion of the tissue (whole Achilles tendon-aponeurosis vs. free Achilles tendon) analysed and imaging methods (2D vs. 3D) used to track tissue deformation. For instance, cadaveric Achilles tendon samples studied by Wren et al.(2001) included a substantial portion of the Achilles tendon aponeurosis; whereas Lewis and Shaw (1997) analysed the free Achilles tendon portion only. It is possible that Wren et al. (2001) may have under-estimated the strain experienced by the free Achilles tendon for a given load and may have over-estimated the Young’s modulus of the samples tested. Nonetheless, we analysed the free Achilles tendon region similar to Lewis and Shaw (1997) and the Young’s modulus values reported in the two studies are similar. Furthermore, the freehand 3DUS approach used to assess free Achilles tendon geometry (volume, length, and CSA) in the present study has been shown to provide high levels of test-retest reliability (intra-class correlation coefficient > 0.98) and accuracy of phantom length measures (standard error = 0.2 mm; Obst et al., 2014a). Consistent with other studies of the Achilles tendon, our results suggest that changes in the free Achilles tendon mechanical stiffness in trained elite/sub-elite middle-distance runners are primarily driven by alterations in free Achilles tendon geometry, rather than changes in Young’s modulus (Wiesinger et al., 2015, 2016).

T2^*^ relaxation times of the mid-portion of the free Achilles tendon were significantly longer in runners compared to non-runners. Longer T2^*^ relaxation time in tendons has been suggested to represent greater amount of free water protons between collagen fibres that could reflect increased water content and/or disorganised collagen alignment (Diaz et al., 2012; Juras et al., 2012, 2013). Our T2^*^ relaxation times were computed using a mono-exponential decay function, and therefore, represent the weighted mean of fast and slow T2^*^ relaxation times (Juras et al., 2013). Therefore, we cannot say whether our results reflect increased content of bound or unbound “free” water molecules, which may be possible using a bi-exponential decay function to measure the fast and slow T2^*^ relaxation times, respectively. Regardless, our results are broadly supported by studies that report longer mono-exponential T2^*^ relaxation times in recreational runners versus non-runners (Grosse et al., 2015), and in individuals with tendon pathology versus healthy controls (Juras et al., 2013; Gärdin et al., 2016). Longer T2^*^ relaxation time in runners is also consistent with findings of Hullfish et al. (2018) who reported the lower collagen alignment and ultrasound echogenicity in runners compared to non-runners. Taken together with the findings of Grosse et al. (2015) and Hullfish et al. (2018), the findings of the present study indicate that the free Achilles tendon of trained elite/sub-elite middle-distance runners exhibits features also reported for pathological tendons (i.e., increased CSA and poorly organised collagen) and appear to place runners on a continuum of Achilles tendon T2^*^ relaxation time that is intermediate between healthy and pathological Achilles tendons. Further, T2^*^ relaxation time has been shown to correlate with clinical scores in Achilles tendinopathy (Juras et al., 2013). The lack of significant correlation between T2^*^ relaxation time and Young’s modulus from the present study suggests that T2^*^ relaxation time should not be considered a valid proxy for the free Achilles tendon Young’s modulus. There are several possible explanations for this finding. Firstly, T2^*^ relaxation time may primarily reflect the hydration state of the tissue, to a greater extent than the degree of collagen disorganisation, and so a greater concentration of free water in runner would not be expected to influence material properties. Alternatively, the extent of collagen disorganisation detected in runners may be below the threshold required to influence material properties of the tendon.

### Limitations

There are several limitations to this study. Firstly, while all MRI scans were obtained on the same model of MRI machine and using the same MRI sequences, the two participant groups were scanned at different facilities, so we cannot fully discount the possibility of a systematic error in T2^*^ relaxation time estimates. Nonetheless, T2^*^ relaxation times reported in the present study are within the range of values reported in the literature (Juras et al., 2012; Grosse et al., 2015) and followed similar trends. Further, the T2^*^ relaxation time reported in this study was the mean value obtained from pixels within the tendon CSA within five image slices (mid-portion of the free Achilles tendon). Future studies should examine regional variation in T2^*^ relaxation time. While we performed free Achilles tendon measurements in carefully controlled conditions, it is still possible that small errors in ankle joint torque measures could be present. The triceps surae muscle forces used to compute mechanical stiffness of the free Achilles tendon were calculated as the ratio between measured ankle plantarflexion moment and moment arm measured during a static MRI. As the measured ankle plantarflexion moment is influenced by the activity of muscles other than the triceps surae (e.g., tibialis anterior), the EMG signals of the triceps surae and tibialis anterior muscles were visually inspected and instructions were provided by the operator to the participant to prevent co-contraction between the triceps surae and tibialis anterior muscles. Furthermore, we ensured that the participant’s foot was firmly secured to the foot plate of dynamometer and the ankle joint axis was aligned with the dynamometer axis. However, some misalignment between the ankle joint axis and dynamometer axis could have been present during some of the high intensity active muscle contractions (70% MVC). We also acknowledge that the two participant groups differed in age on average by 5 years and were not equally balanced by gender and that this may had a small influence on our overall findings. Finally, given the cross-sectional design of the study, it is not possible to determine whether the observed differences in free Achilles tendon properties reflect training adaptations or are to some extent pre-existing properties that favour running performance. Prospective studies are required to address this question. Modelling studies that account for an individual’s free Achilles tendon properties are also required to understand the effects of training induced tendon adaptations on triceps surae fascicle behaviour and running economy.

## Conclusion

The free Achilles tendon of trained elite/sub-elite middle-distance runners is stiffer than healthy controls primarily due to an altered geometry. In the absence of group differences in tendon material properties, the larger free Achilles tendon CSA in runners compared to healthy controls appears to be the primary mechanism that protects the free Achilles tendon of runners against high stresses and strains. Although T2^*^ relaxation time is sufficiently sensitive to detect differences between trained runners and healthy controls, T2^*^ relaxation time should not be considered a valid proxy for Achilles tendon material properties.

## Data Availability Statement

The raw data supporting the conclusions of this article will be made available by the authors upon reasonable request.

## Ethics Statement

The studies involving human participants were approved by the Human Research Ethics Committee’s at Griffith University and the Australian Institute of Sport. The participants provided their written informed consent to participate in this study.

## Author Contributions

DD, SO, DL, RB, BK, IB, NV, AH, DP, and CP conceived and designed the research. DD, CP, SJO, BK, IB, KA, TC, GD, AH, DP, and NV conducted the experiments. DD, CP, SO, RB, and DL analysed and interpreted the data. DD, SO, RB, and CP wrote the initial manuscript draft. All authors contributed to the article and approved the submitted version.

### Conflict of Interest

IB was employed by the company Philips Healthcare, Australia and New Zealand.The remaining authors declare that the research was conducted in the absence of any commercial or financial relationships that could be construed as a potential conflict of interest.
